# Supplementary material for: Tissue-specific consequences of tag fusions on protein expression in transgenic mice
Source: PLoS Genet. 2025 Aug 25;21(8):e1011830. doi: 10.1371/journal.pgen.1011830 (PMC12407551; doi:10.1371/journal.pgen.1011830)
Supplement: S1 Fig — A. Western blot showing the bands detected by anti-NCAPH2 antibody from whole adult brain lysates of animals with 0, 1 or 2 alleles of the C-terminal AID:Clover tag fusion. N.S. indicates the position of non-specific band. B. Western blots on lysates of thymocytes from Rosa26Tir1/Tir1 homozygotes either homozygous (Tg/Tg) or wildtype (+/+) for the Ncaph2AID:Clover allele cultured ex vivo for 3 hours in the presence or absence of 500 μM indole-3-acetic acid (IAA). C. Western blots of NIH-3T3 cell lysates, harvested 48 hours following transfection with an siRNA pool directed against mouse Ncaph2. Intensities of wildtype and non-specific bands were quantified relative to GAPDH over n = 2 independent transfections. (PDF) [file pgen.1011830.s001.pdf]

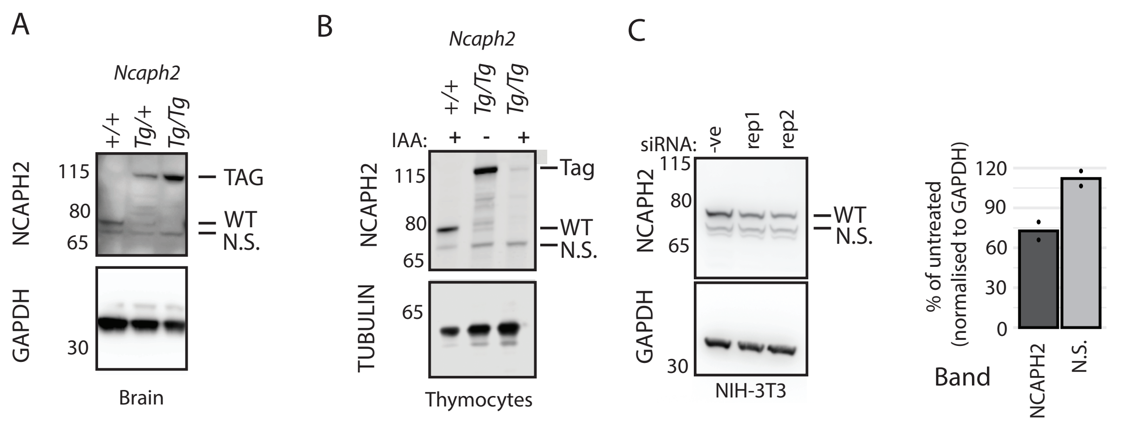

S1 Fig : A ~65 KDa band detected by an anti-NCAPH2 antibody is not produced by the *Ncaph2* gene.

A. Western blot showing the bands detected by anti-NCAPH2 antibody from whole adult brain lysates of animals with 0, 1 or 2 alleles of the C-terminal AID:Clover tag fusion. N.S. indicates the position of non-specific band. **B.** Western blots on lysates of thymocytes from *Rosa26*$^{Tir1/Tir1}$ homozygotes either homozygous (Tg/Tg) or wildtype (+/+) for the *Ncaph2*$^{AID:Clover}$ allele cultured *ex vivo* for 3 hours in the presence or absence of 500 μM indole-3-acetic acid (IAA). **C.** Western blots of NIH-3T3 cell lysates, harvested 48 hours following transfection with an siRNA pool directed against mouse *Ncaph2*. Intensities of wildtype and non-specific bands were quantified relative to GAPDH over n = 2 independent transfections.
