# Supplementary material for: Tissue-specific consequences of tag fusions on protein expression in transgenic mice
Source: PLoS Genet. 2025 Aug 25;21(8):e1011830. doi: 10.1371/journal.pgen.1011830 (PMC12407551; doi:10.1371/journal.pgen.1011830)
Supplement: S2 Fig — A. Schematic showing the C-terminal APEX2 fusion at Ift81. B. Western blots on whole tissue lysates from adult animals heterozygous (left) or wildtype (right) for Ift81APEX2. The tag causes a band shift of 28 kDa. The protein is expressed at highest levels in motile ciliated cells which are abundant in the testes and trachea, where the ratio is approximately 1:1. At longer exposure times multiple bands of variable sizes were observed in wildtype and heterozygous samples from other tissues. Since there are no described isoforms for IFT81, we assume these are non-specific bands. C. Schematic showing the N-terminal auxin-inducible degron (AID) fusion at Ddx3x. D. Western blots on tissue lysates from adult female mice heterozygous for Ddx3xAID. These mice did not co-express the TIR1 protein. Wildtype DDX3X is 73 kDa, and the AID tag causes a band shift of 7 kDa, which was insufficient to enable reliable individual quantification of tagged and wildtype bands. Western blot is representative of n = 3 biological replicates. (PDF) [file pgen.1011830.s002.pdf]

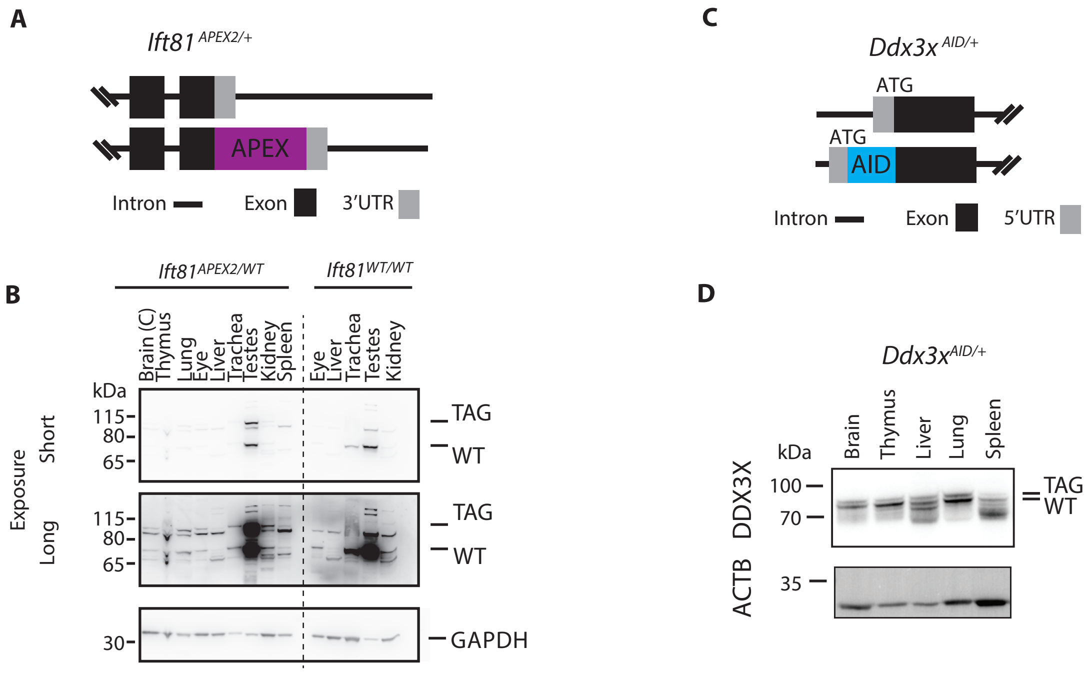

S2 Fig : Multi-tissue expression of tagged DDX3X and IFT81 proteins

A. Schematic showing the C-terminal APEX2 fusion at *Ift81*. **B**. Western blots on whole tissue lysates from adult animals heterozygous (left) or wildtype (right) for *Ift81^{APEX2}*. The tag causes a band shift of 28 kDa. The protein is expressed at highest levels in motile ciliated cells which are abundant in the testes and trachea, where the ratio is approximately 1:1. At longer exposure times multiple bands of variable sizes were observed in wildtype and heterozygous samples from other tissues. Since there are no described isoforms for IFT81, we assume these are non-specific bands. **C.** Schematic showing the N-terminal auxin-inducible degron (AID) fusion at *Ddx3x*. **D.** Western blots on tissue lysates from adult female mice heterozygous for *Ddx3x^{AID}*. These mice did not co-express the TIR1 protein. Wildtype DDX3X is 73 kDa, and the AID tag causes a band shift of 7 kDa, which was insufficient to enable reliable individual quantification of tagged and wildtype bands. Western blot is representative of n = 3 biological replicates.
