# Supplementary material for: Tissue-specific consequences of tag fusions on protein expression in transgenic mice
Source: PLoS Genet. 2025 Aug 25;21(8):e1011830. doi: 10.1371/journal.pgen.1011830 (PMC12407551; doi:10.1371/journal.pgen.1011830)
Supplement: S3 Fig — A. Schematic illustrating the design of allele-specific qRT-PCR assays to measure transcript ratios in tissues from Ncaph2AID:Clover/+ and NcaphAID:Clover (left) and Sox2Halo (right). B. Histogram showing ΔΔCt values, expressed as a ratio of tagged/ wildtype gene product from n = 3 biological replicate samples per tissue for Ncaph2 and Sox2, n = 4 samples for Ncaph. (PDF) [file pgen.1011830.s003.pdf]

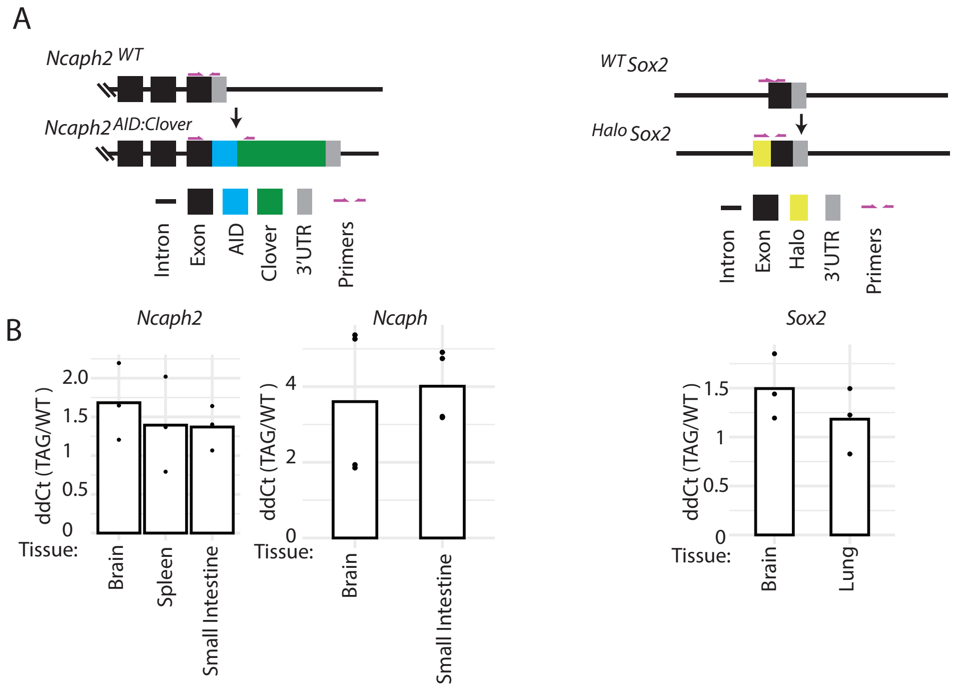

S3 Fig : The ratio of tagged to untagged mRNA is not significantly different across tissues for *Ncaph2*, *Ncaph* and *Sox2*.
A. Schematic illustrating the design of allele-specific qRT-PCR assays to measure transcript ratios in tissues from *Ncaph2*$^{AID:Clover/+}$ and *Ncaph*$^{AID:Clover}$ (left) and *Sox2*^{Halo} (right). **B.** Histogram showing ΔΔCt values, expressed as a ratio of tagged / wildtype gene product from n = 3 biological replicate samples per tissue for *Ncaph2* and *Sox2*, n = 4 samples for *Ncaph*.
