# Supplementary material for: Tissue-specific consequences of tag fusions on protein expression in transgenic mice
Source: PLoS Genet. 2025 Aug 25;21(8):e1011830. doi: 10.1371/journal.pgen.1011830 (PMC12407551; doi:10.1371/journal.pgen.1011830)
Supplement: S4 Fig — Boxplots show degradation of NCAPH2AID:Clover induced by 2 hour exposure of primary thymocytes to 100μM IAA. Degradation was inhibited by addition of either 10μM MG132 (A) or 5μM MLN4924 (B), indicating successful inhibition of the proteasome, or Cullin RING Ubiquitin ligases, respectively. (PDF) [file pgen.1011830.s004.pdf]

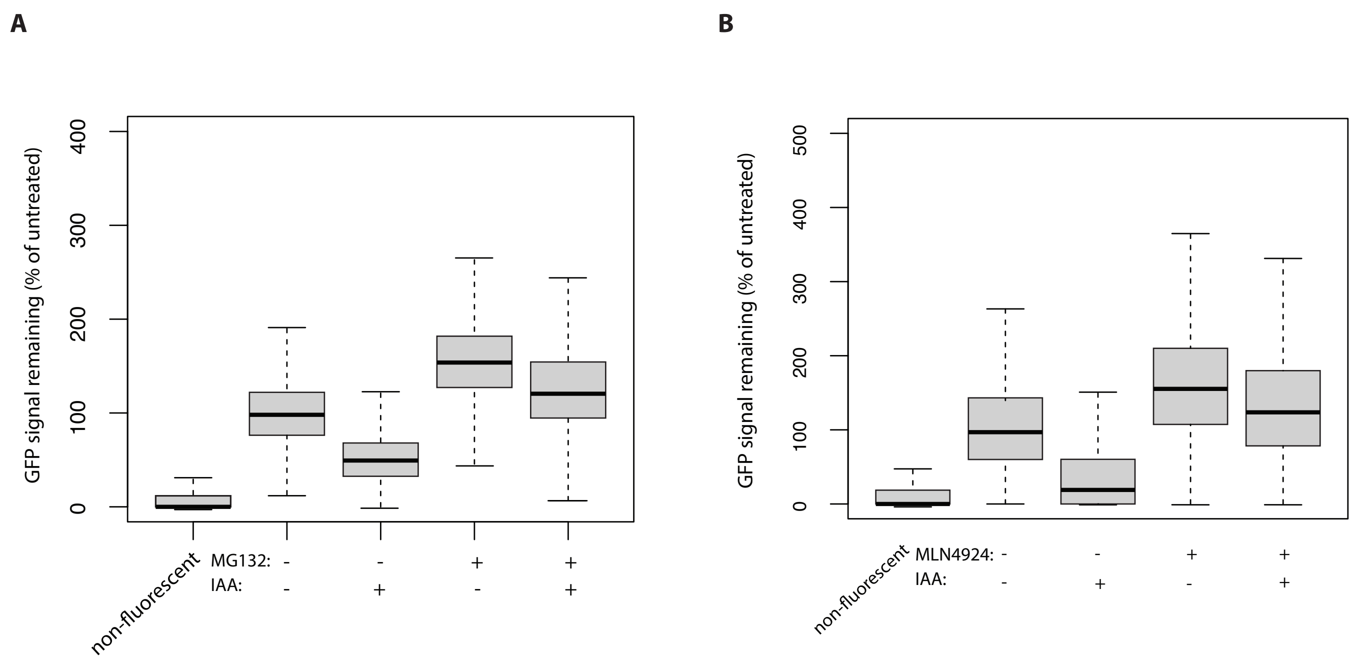

S4 Fig : Control experiments show efficacy of MG132 and MLN4924 compounds.
Boxplots show degradation of NCAPH2$^{AID:Clover}$ induced by 2 hour exposure of primary thymocytes to 100µM IAA. Degradation was inhibited by addition of either 10µM MG132 (**A**) or 5µM MLN4924 (**B**), indicating successful inhibition of the proteasome, or Cullin RING Ubiquitin ligases, respectively.
