# Supplementary material for: Tissue-specific consequences of tag fusions on protein expression in transgenic mice
Source: PLoS Genet. 2025 Aug 25;21(8):e1011830. doi: 10.1371/journal.pgen.1011830 (PMC12407551; doi:10.1371/journal.pgen.1011830)

Figure 1B

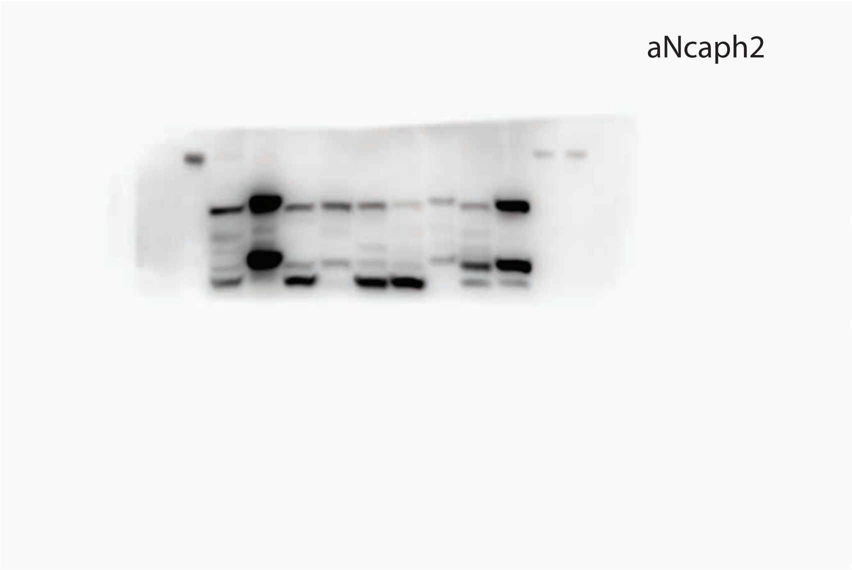

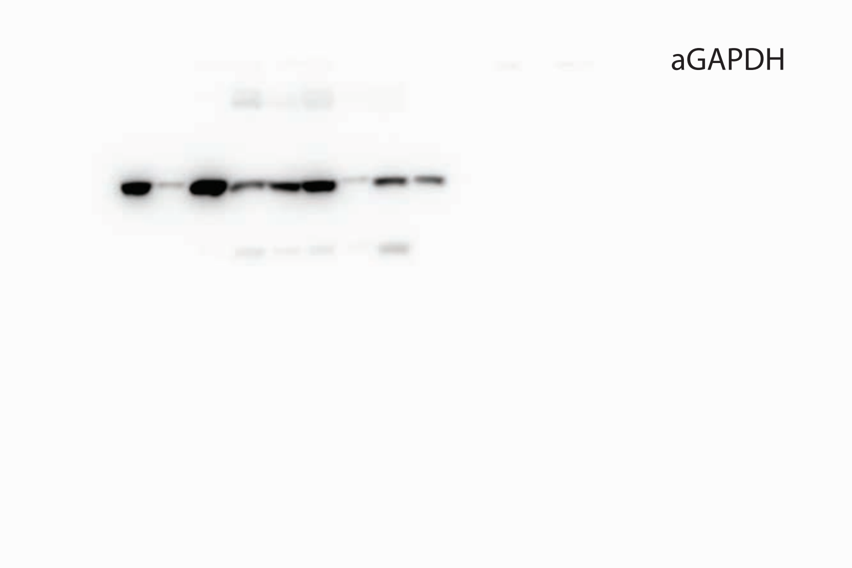

Figure 1C

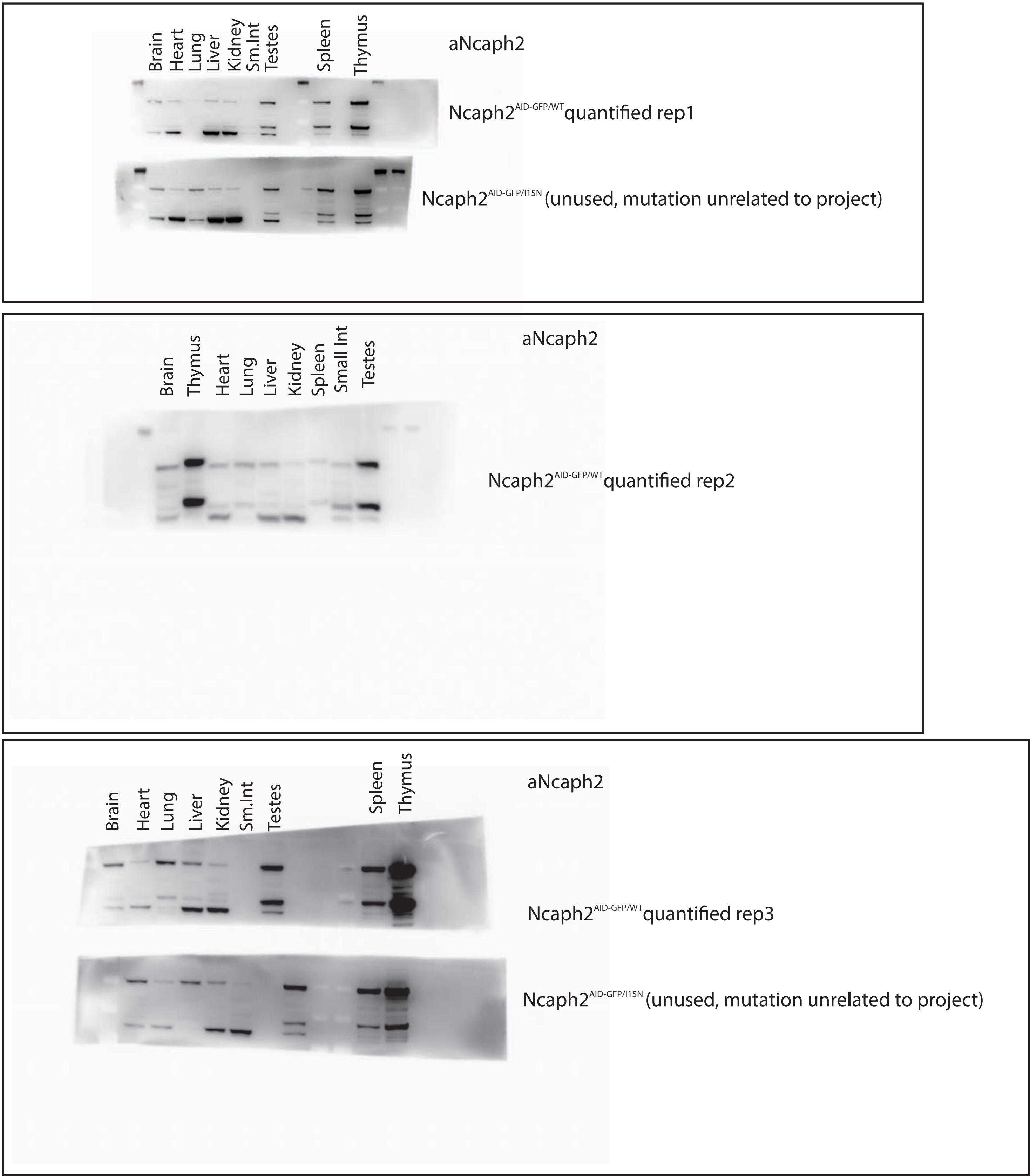
Brain
Heart
Lung
Liver
Kidney
Sm.Int
Testes
Spleen
Thymus
aNcaph2
Ncaph2AID-GFP/WT quantified rep1
Ncaph2AID-GFP/I15N (unused, mutation unrelated to project)
Brain
Thymus
Heart
Lung
Liver
Kidney
Spleen
Small Int
Testes
aNcaph2
Ncaph2AID-GFP/WT quantified rep2
Brain
Heart
Lung
Liver
Kidney
Sm.Int
Testes
Spleen
Thymus
aNcaph2
Ncaph2AID-GFP/WT quantified rep3
Ncaph2AID-GFP/I15N (unused, mutation unrelated to project)

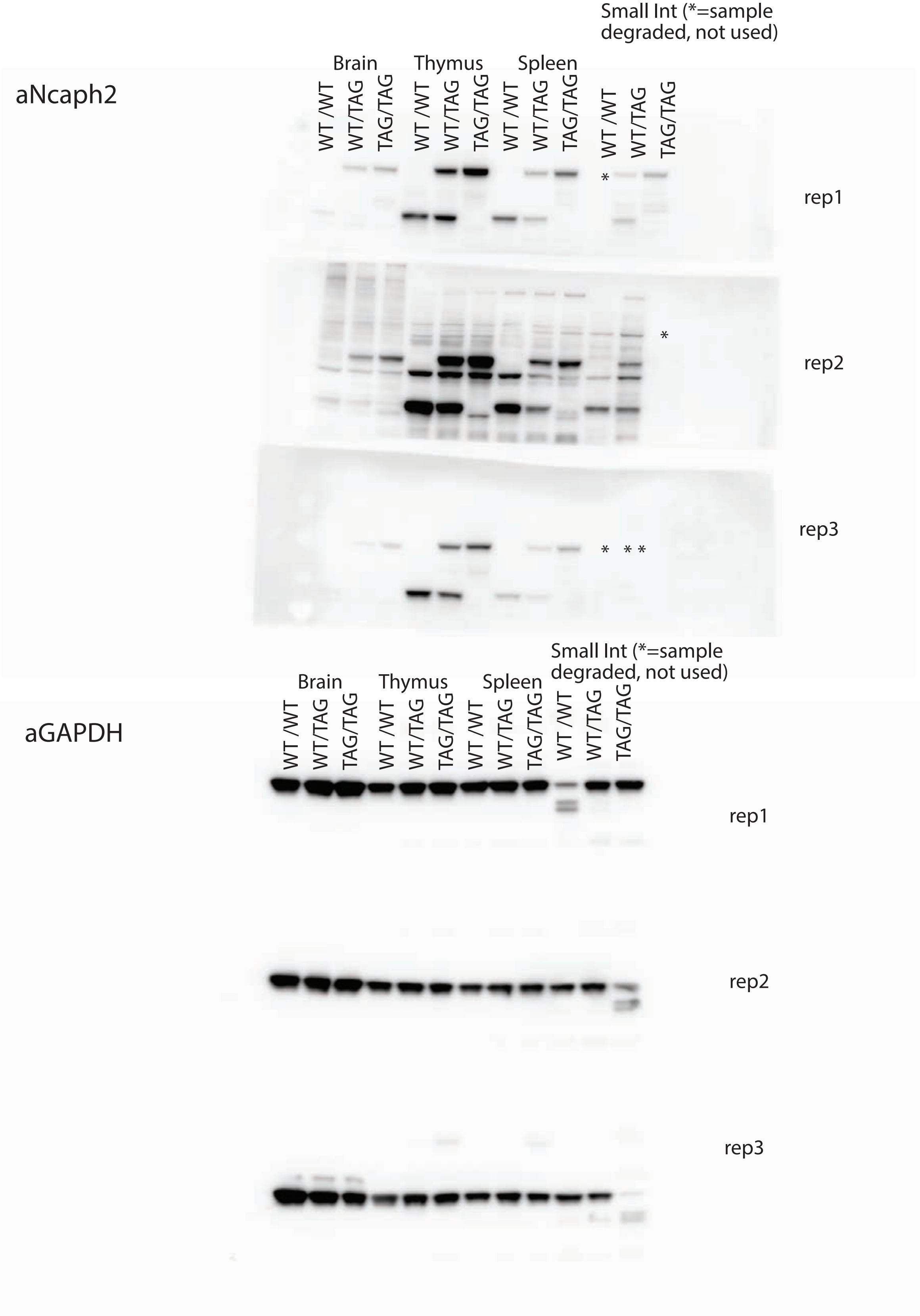
aNcaph2
Brain
Thymus
Spleen
Small Int (*=sample degraded, not used)
WT /WT
WT/TAG
TAG/TAG
WT /WT
WT/TAG
TAG/TAG
WT /WT
WT/TAG
TAG/TAG
WT /WT
WT/TAG
TAG/TAG
*
rep1
*
rep2
* * *
rep3
aGAPDH
Brain
Thymus
Spleen
Small Int (*=sample degraded, not used)
WT /WT
WT/TAG
TAG/TAG
WT /WT
WT/TAG
TAG/TAG
WT /WT
WT/TAG
TAG/TAG
WT /WT
WT/TAG
TAG/TAG
rep1
rep2
rep3

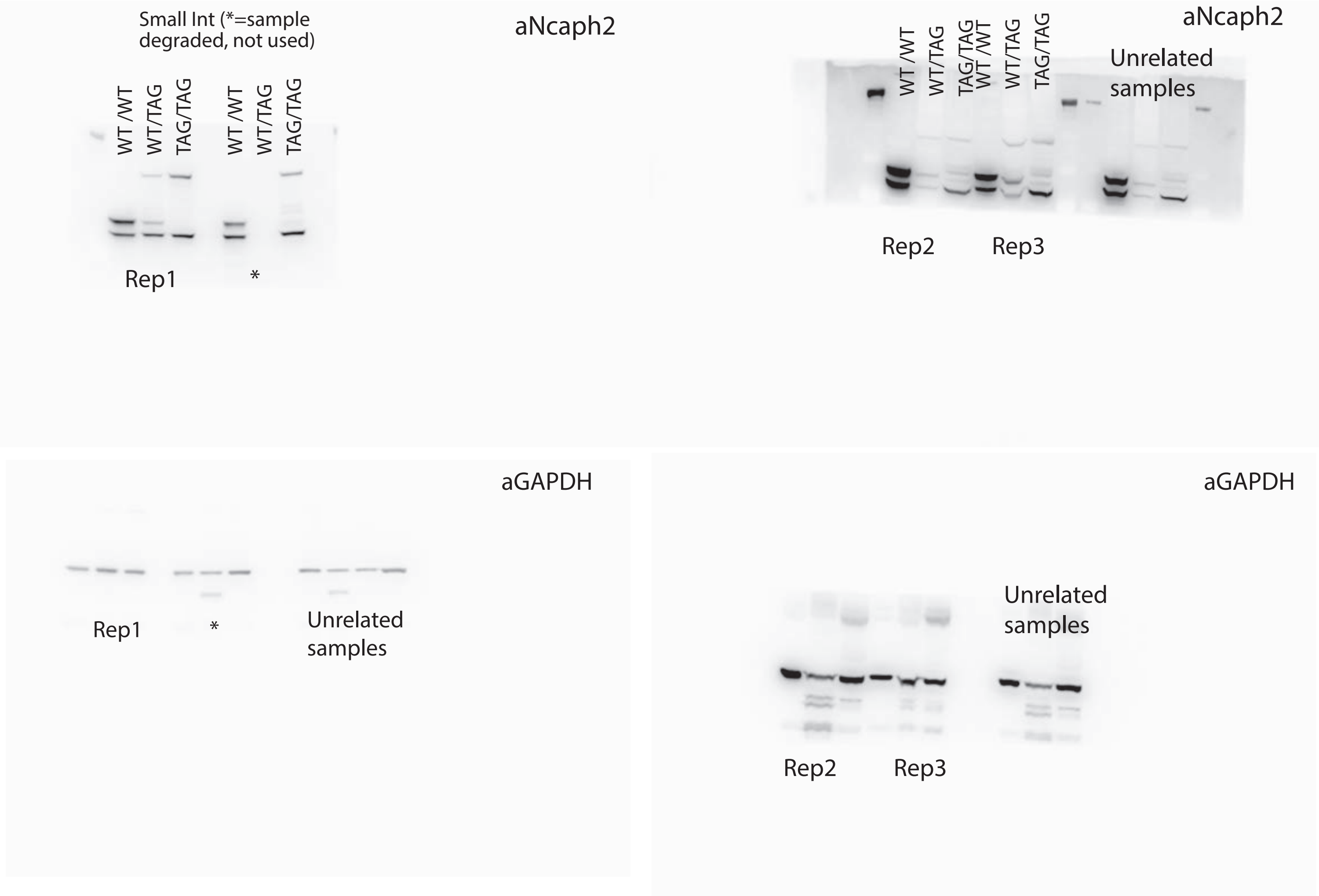
Small Int (*=sample degraded, not used)
aNcaph2
WT /WT
WT/TAG
TAG/TAG
WT /WT
WT/TAG
TAG/TAG
Rep1
*
aNcaph2
WT /WT
WT/TAG
TAG/TAG
WT /WT
WT/TAG
TAG/TAG
Unrelated samples
Rep2
Rep3
aGAPDH
Rep1
*
Unrelated samples
aGAPDH
Unrelated samples
Rep2
Rep3

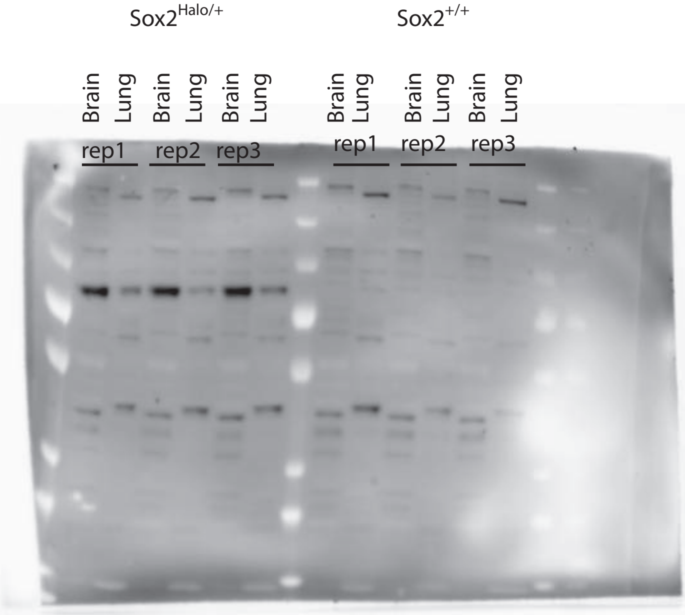

Sox2$^{Halo/+}$
Sox2$^{+/+}$
Brain
Lung
Brain
Lung
Brain
Lung
Brain
Lung
Brain
Lung
Brain
Lung
rep1
rep2
rep3
rep1
rep2
rep3

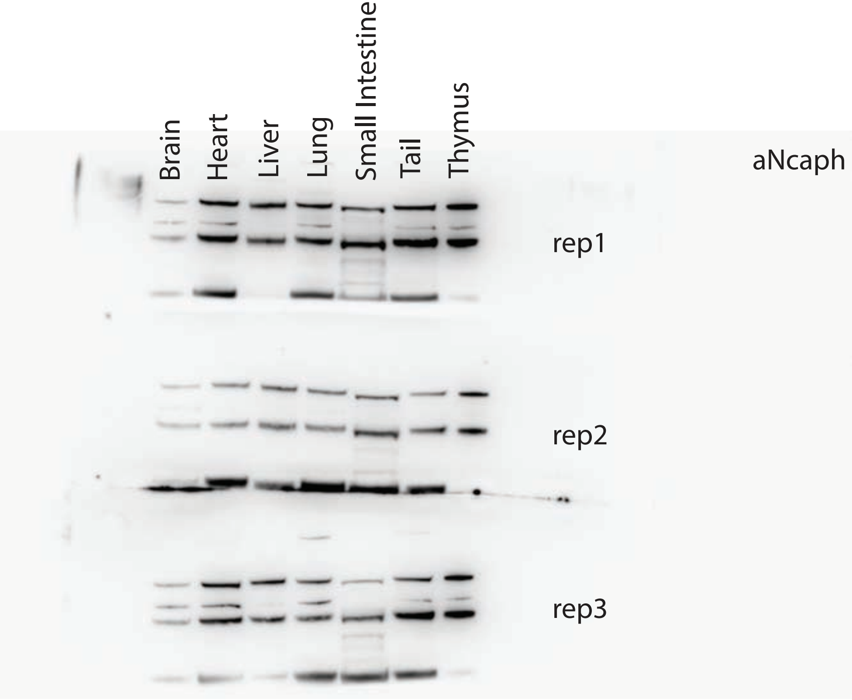
Brain
Heart
Liver
Lung
Small Intestine
Tail
Thymus
aNcaph
rep1
rep2
rep3

Small Intestine (*=sample degraded, not used)

Thymus

-/- Tir1/Tir1 -/- Tir1/Tir1 -/- Tir1/Tir1 -/- Tir1/Tir1 -/- Tir1/Tir1

Rosa26:

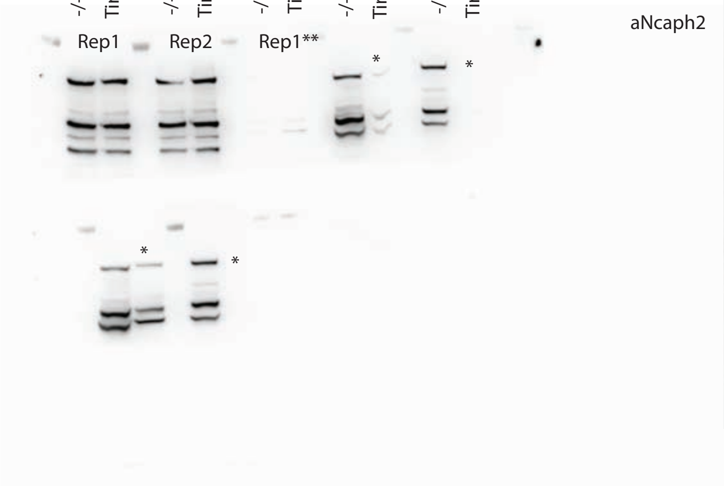

noOsTIR1-Rep4
Tir1/Tir1-Rep1

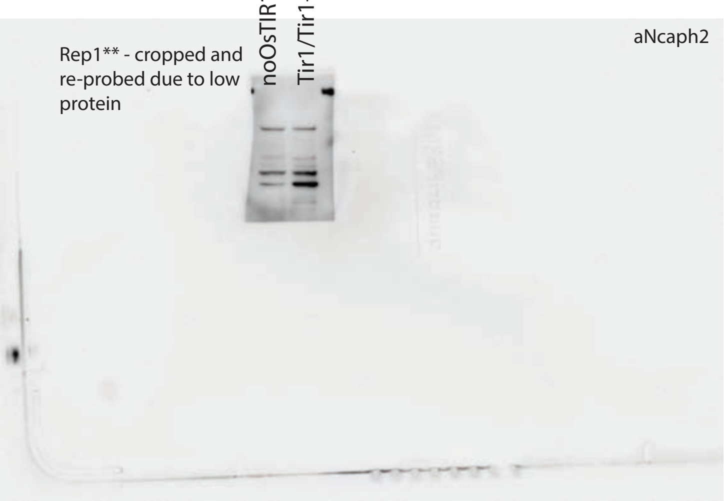

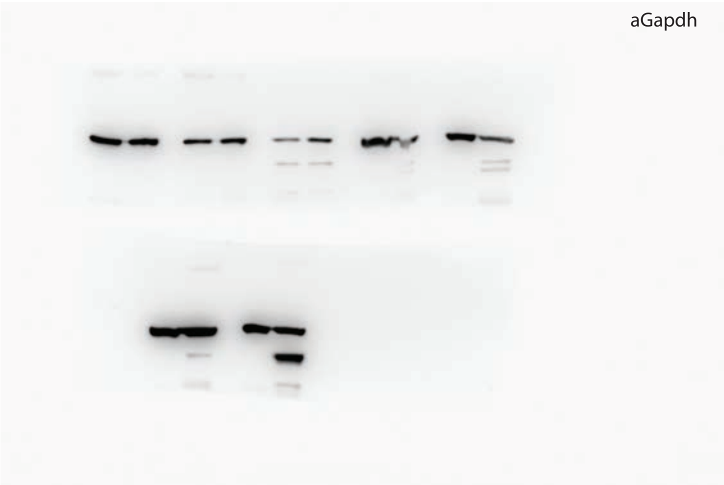

Tir1/Tir1-Rep2
Tir1/Tir1-Rep3

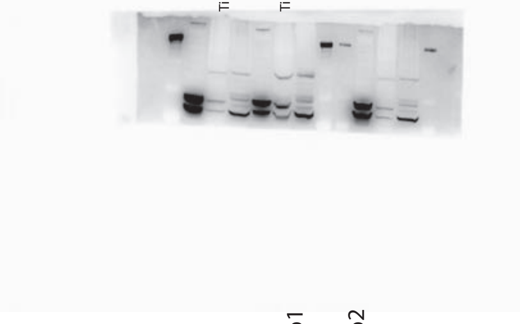

Tir1/Tir1-Rep4

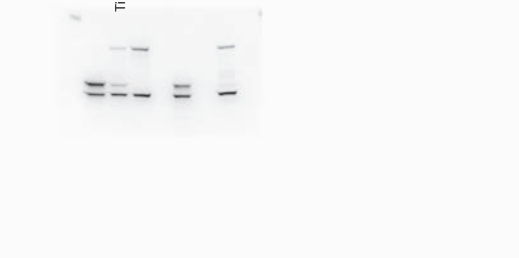

Tir1/Tir1-Rep5

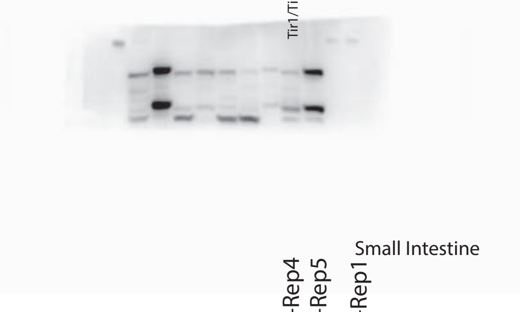

Small Intestine

noOsTIR1-Rep1
noOsTIR1-Rep2

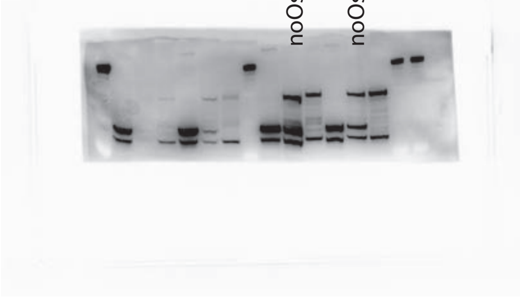

noOsTIR1-Rep3

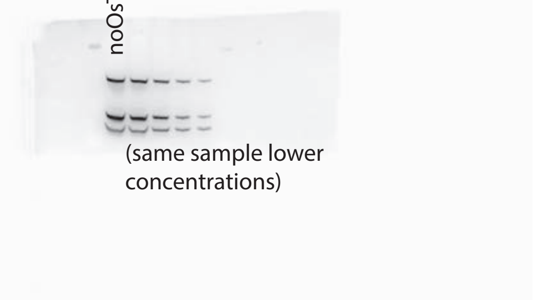

(same sample lower concentrations)

noOsTIR1-Rep4
noOsTIR1-Rep5
Tir1/Tir1-Rep1

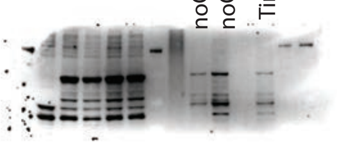

Small Intestine

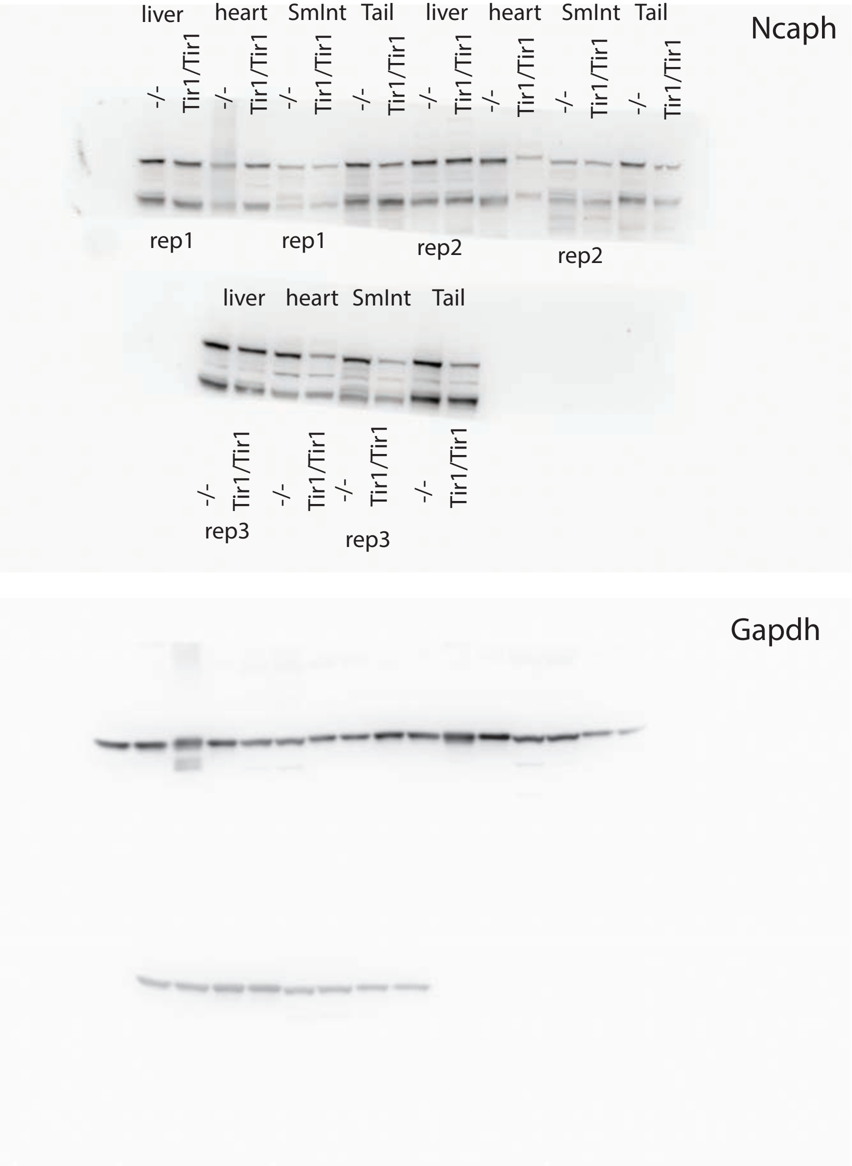
Ncaph
liver heart SmInt Tail liver heart SmInt Tail
-/- Tir1/Tir1 -/- Tir1/Tir1 -/- Tir1/Tir1 -/- Tir1/Tir1 -/- Tir1/Tir1 -/- Tir1/Tir1 -/- Tir1/Tir1 -/- Tir1/Tir1
rep1 rep1 rep2 rep2
liver heart SmInt Tail
-/- Tir1/Tir1 -/- Tir1/Tir1 -/- Tir1/Tir1 -/- Tir1/Tir1
rep3 rep3
Gapdh

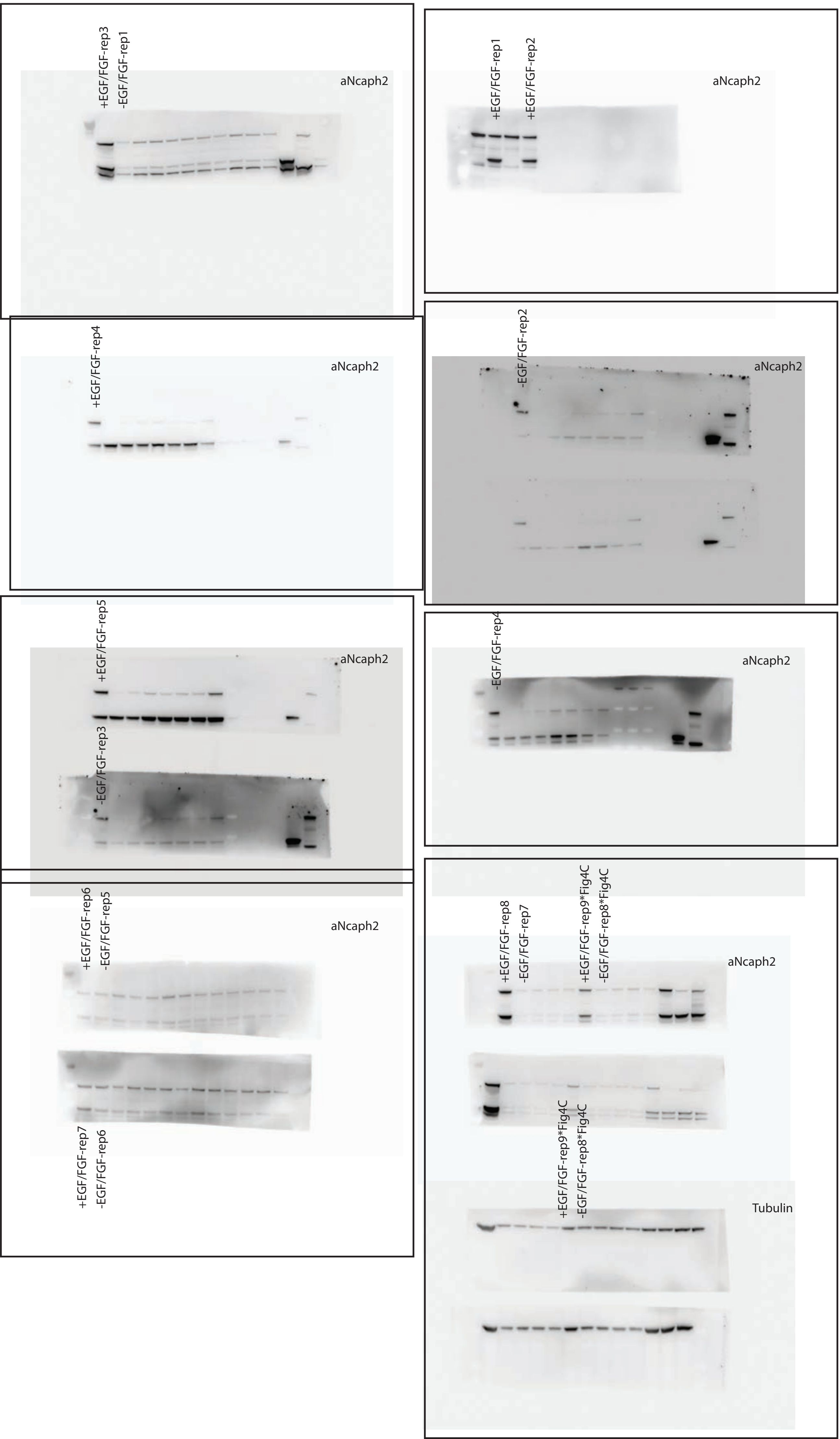
+EGF/FGF-rep3
-EGF/FGF-rep1
aNcaph2
+EGF/FGF-rep1
+EGF/FGF-rep2
aNcaph2
+EGF/FGF-rep4
aNcaph2
-EGF/FGF-rep2
aNcaph2
+EGF/FGF-rep5
aNcaph2
-EGF/FGF-rep3
-EGF/FGF-rep4
aNcaph2
+EGF/FGF-rep6
-EGF/FGF-rep5
aNcaph2
+EGF/FGF-rep7
-EGF/FGF-rep6
+EGF/FGF-rep8
-EGF/FGF-rep7
+EGF/FGF-rep9*Fig4C
-EGF/FGF-rep8*Fig4C
aNcaph2
+EGF/FGF-rep9*Fig4C
-EGF/FGF-rep8*Fig4C
Tubulin

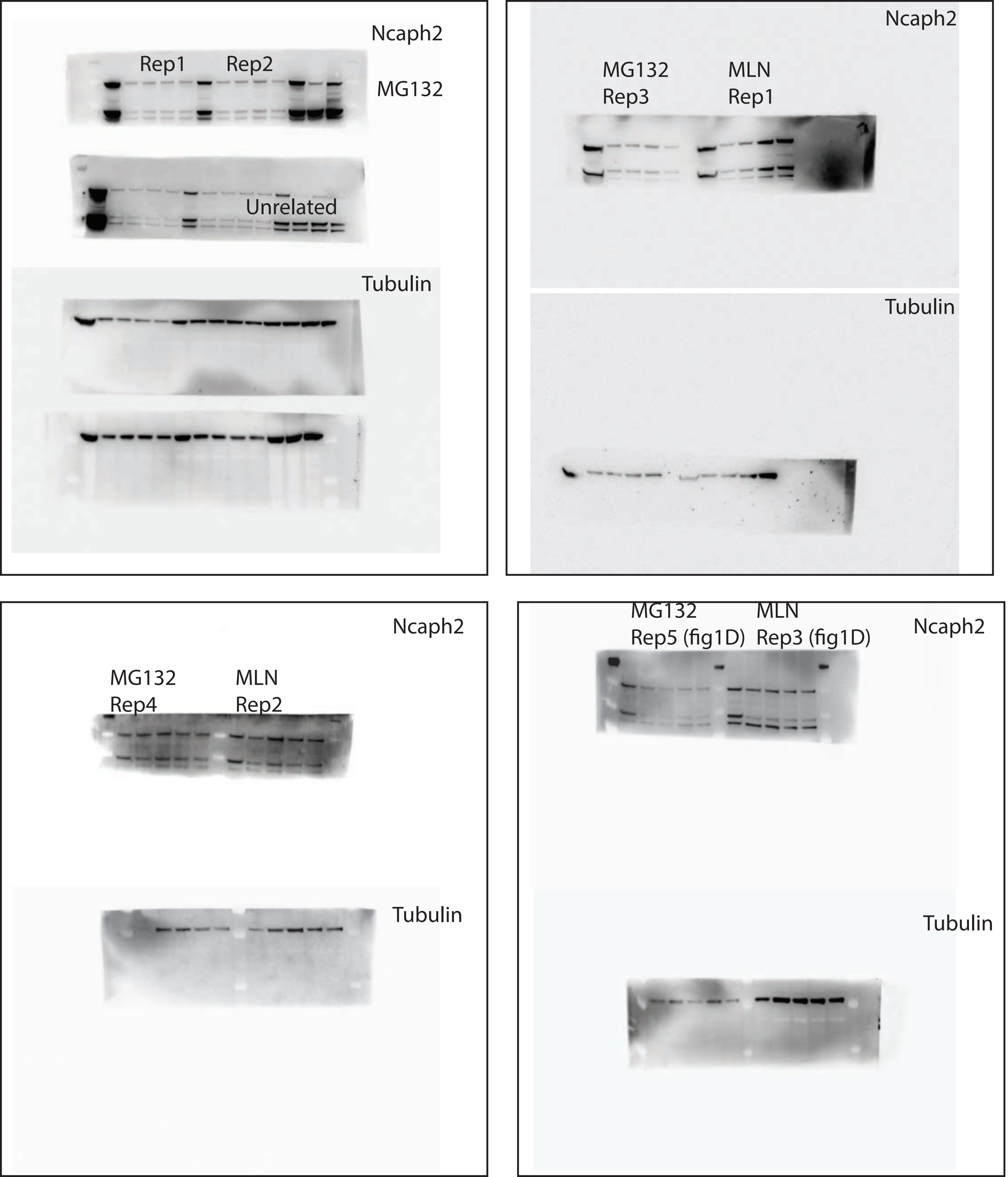
Ncaph2
Rep1
Rep2
MG132
Unrelated
Tubulin
Ncaph2
MG132
Rep3
MLN
Rep1
Tubulin
Ncaph2
MG132
Rep4
MLN
Rep2
Tubulin
MG132
Rep5 (fig1D)
MLN
Rep3 (fig1D)
Ncaph2
Tubulin

Figure 5C

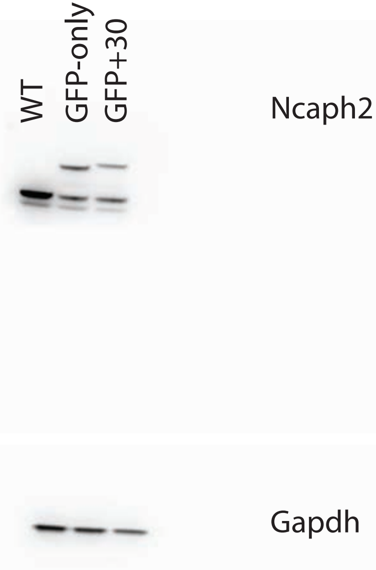

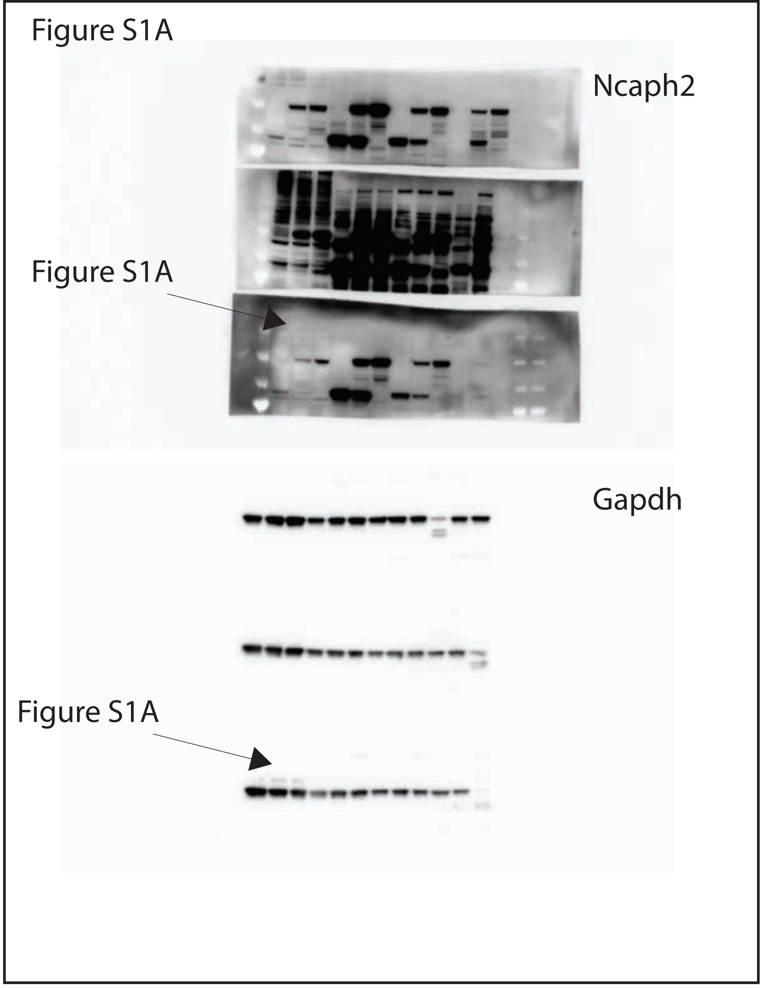
Figure S1A
Ncaph2
Figure S1A
Gapdh
Figure S1A

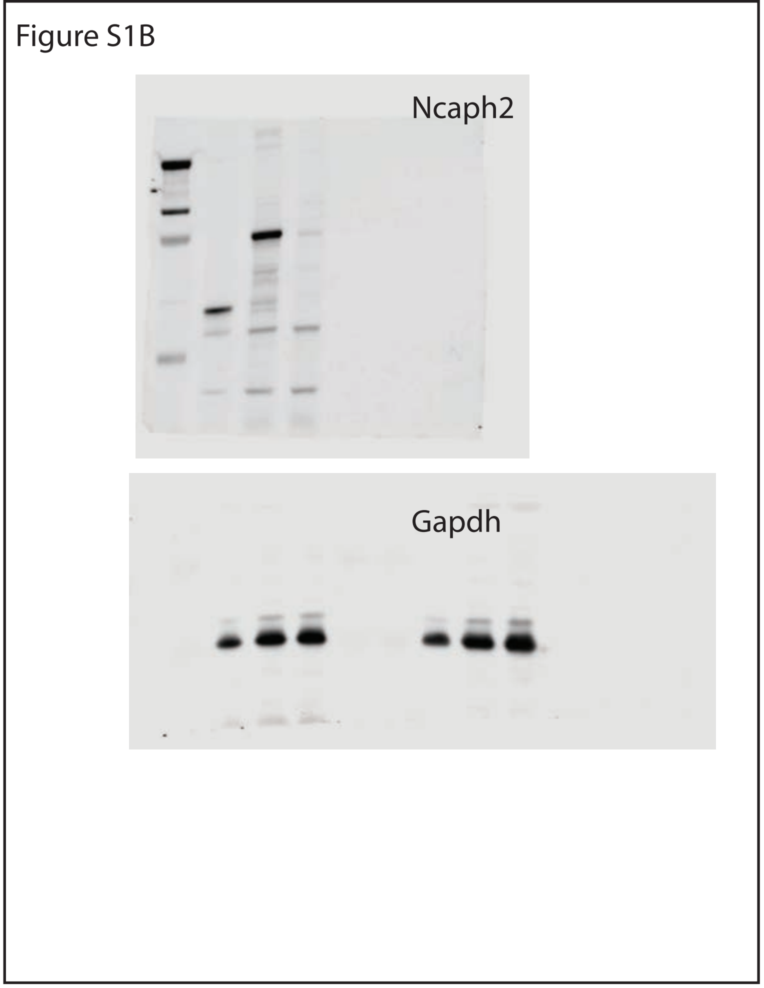
Figure S1B
Ncaph2
Gapdh

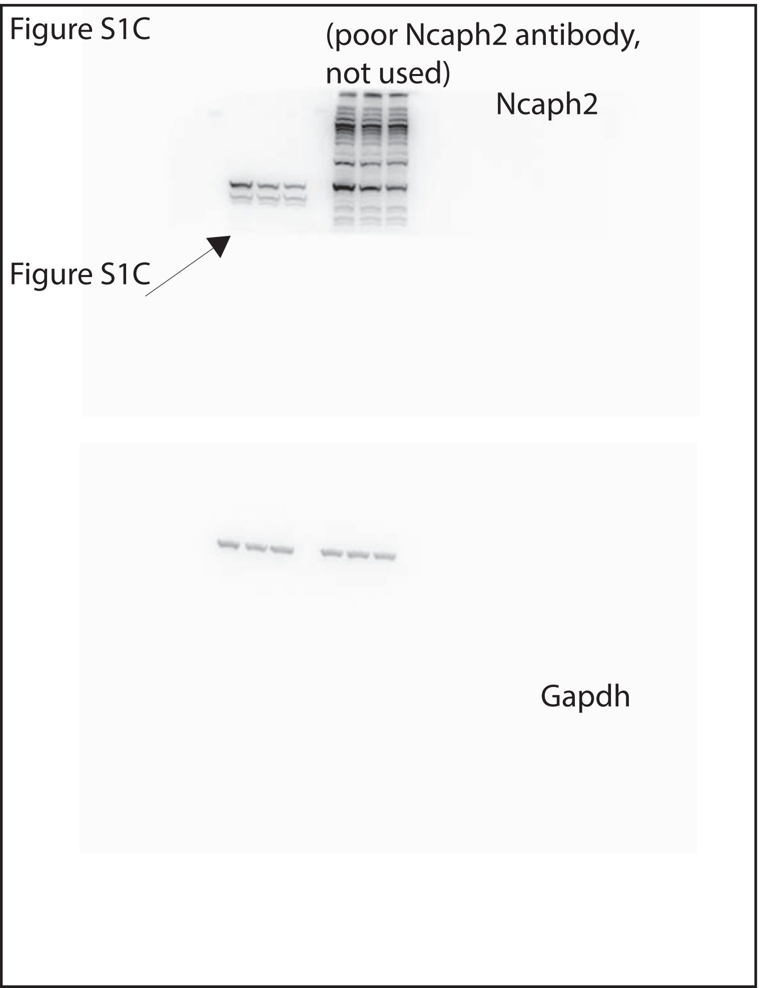
Figure S1C
(poor Ncaph2 antibody, not used)
Ncaph2
Figure S1C
Gapdh

FigureS2A

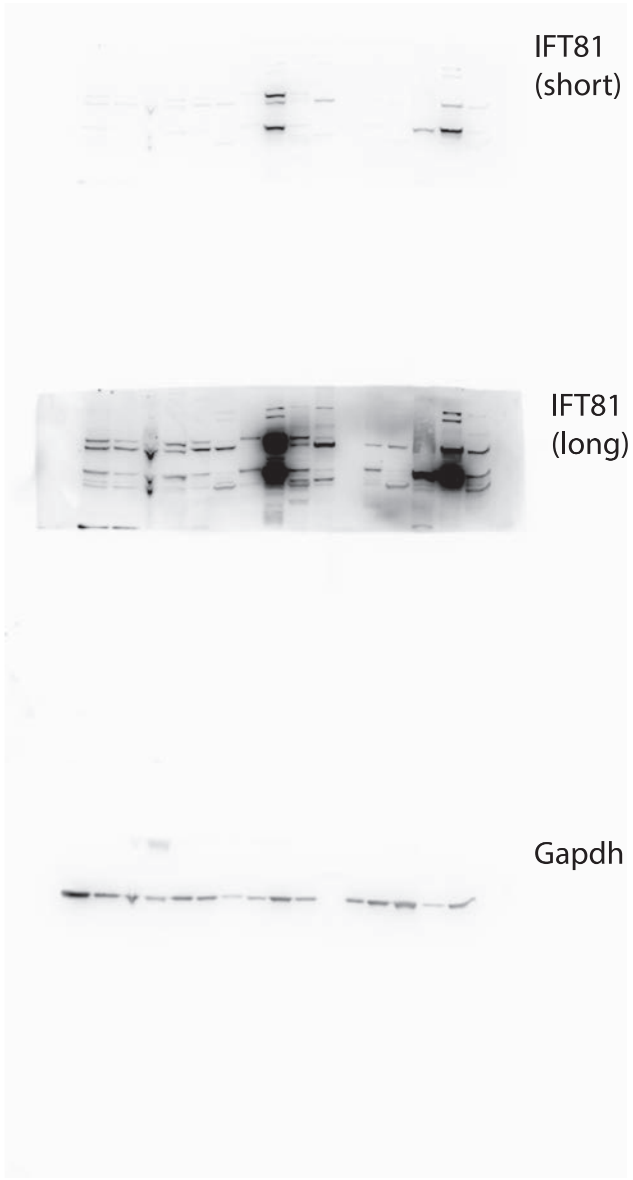

FigureS2B

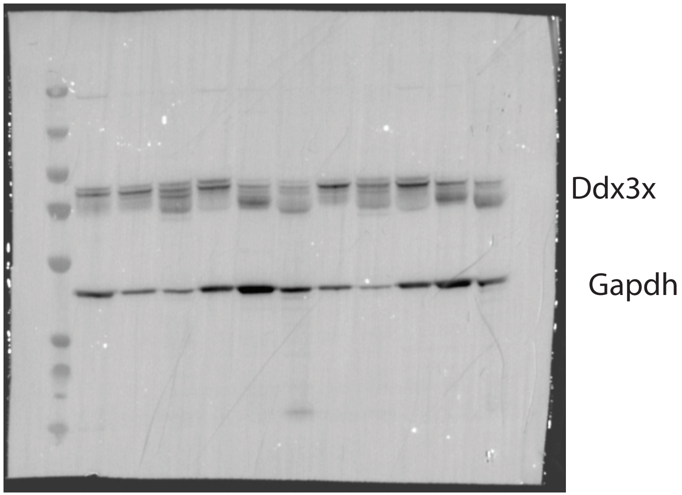

Supplement: S1 File — Image files for western blots used for protein quantification throughout the manuscript. (PDF) [file pgen.1011830.s008.pdf]
