# Supplementary material for: Tissue-specific consequences of tag fusions on protein expression in transgenic mice
Source: PLoS Genet. 2025 Aug 25;21(8):e1011830. doi: 10.1371/journal.pgen.1011830 (PMC12407551; doi:10.1371/journal.pgen.1011830)
Supplement: Attachment — Submitted filename: Reviewer Response.docx [file pgen.1011830.s009.docx]

**Response to Reviewers**

We would like to thank the three reviewers for their positive and constructive comments on our manuscript. Their suggestions for additional experiments and clarifications have helped us to improve the work. Our responses to their specific concerns are highlighted in red text below.

**--------------------------------------------------------------------------------------------------------------**

Reviewer #1: Review of "Tissue-specific consequences of tag fusions on protein expression in transgenic mice"
Manuscript Number: PGENETICS-D-25-00446
Overall Assessment: This manuscript by Taylor et al. investigates the often-underappreciated impact of protein tag fusions on protein expression levels in a tissue-specific manner within transgenic mice. The authors compellingly demonstrate that the consequences of tagging a protein can vary significantly across different tissues and cellular contexts, moving beyond observations typically made in cultured cell lines. They identify two distinct molecular mechanisms underlying these context-dependent effects: tissue-specific "leakage" of the auxin-inducible degron (AID) system due to endogenous auxin levels (particularly in the small intestine), and the disruption of an endogenous C-terminal degron leading to protein stabilization in post-mitotic cells (exemplified by Ncaph2). The study is well-designed, employing heterozygous animals for internal controls and providing robust biochemical evidence. The findings are significant for the broad community of researchers using tagged proteins in vivo, offering valuable insights for experimental design and data interpretation, and suggesting strategies to mitigate these issues.
Main Claims and Significance:
• Main Claims:
1. The effect of tag fusions on protein expression levels can be highly tissue-specific in transgenic mice.
2. C-terminal fusion of an AID:Clover tag to Ncaph2 can lead to increased protein stability in some tissues (e.g., brain) and decreased stability in others (e.g., small intestine).
3. Decreased expression of AID-tagged proteins in the small intestine is due to TIR1-dependent "leakage" of the degron system, likely triggered by basal levels of endogenous auxin/indole-3-acetic acid (IAA) in this tissue.
4. Increased stability of C-terminally tagged Ncaph2 in post-mitotic cells results from the inactivation of an endogenous degradation signal located at the Ncaph2 C-terminus, which is normally active upon cell cycle exit.
5. The authors propose strategies to mitigate these issues, such as using the AID2 system (with Tir1F74G) to reduce leakage and duplicating the endogenous protein terminus to preserve normal degradation signals.

• Significance: These claims are highly significant for the discipline. Genetic tagging is a cornerstone of modern molecular biology, and this work provides crucial caveats and practical guidance for its application in complex animal models. Understanding and mitigating tissue-specific artifacts is vital for the accurate interpretation of protein function studies in vivo and for upholding the 3Rs principles in animal research. The elucidation of specific mechanisms offers a rational basis for improved experimental design.

Novelty and Literature Context:
• Novelty: While it is known that tags can alter protein stability, this study's strength lies in its systematic demonstration and mechanistic investigation of tissue-specific variability in these effects within mouse models. The clear demonstration of endogenous IAA levels causing AID system leakage in a specific tissue (small intestine) in mice is a novel and important finding for users of this popular system. Similarly, the identification of a C-terminal degron in Ncaph2 whose function is masked by tagging and is active in post-mitotic cells, along with the proposed solution of terminal duplication, adds a new dimension to understanding tag interference.
• Literature Context: The authors have appropriately placed their claims within the context of previous literature. They acknowledge prior work on tag-induced protein alterations, the utility of endogenous tagging, the existence of N- and C-terminal degrons, and the development of inducible degron systems. The discussion fairly integrates their findings with existing knowledge, such as reports of AID leakage in other systems and the benefits of the AID2 system.

Data and Analyses:
The data and analyses presented generally provide strong support for the claims:
• Figures 1 and 2 clearly illustrate tissue-specific differences in tagged versus untagged protein ratios for Ncaph2, Sox2, and Ncaph using Western blotting in heterozygous animals.
• Figure 3 robustly supports the AID leakage mechanism in the small intestine by:
o Showing physiologically relevant IAA concentrations in the mouse ileum (from cited data).
o Demonstrating IAA-induced degradation of a reporter in cultured cells at similar concentrations.
o Showing Tir1-dependence of reduced tagged protein levels in the small intestine for both Ncaph2AID:Clover and NcaphAID:Clover.
o Quantifying the reduced IAA sensitivity of the Tir1F74G variant.
• Figure 4 supports the C-terminal degron hypothesis for Ncaph2 by showing:
o Increased tagged/wildtype protein ratio upon cell cycle exit in NSCs.
o Attenuation of wildtype Ncaph2 downregulation by proteasome inhibition (MG132).
• Figure 5 directly tests the C-terminal degron hypothesis by showing that duplicating the terminal 30 amino acids of Ncaph2 after a GFP tag restores its downregulation upon differentiation in mESCs.
• The allele-specific transcript quantification (Figure S3) effectively rules out transcriptional effects as the primary cause of the observed protein level differences.
• The use of heterozygous animals to provide internal controls for protein expression is a strong feature of the study design.

**We are very grateful to the reviewer for their thorough and generous summary of our manuscript.**

Suggestions for Improvement/Additional Work: The study is comprehensive and the conclusions are well-supported.
• While the authors discuss the potential for N-terminal degrons to be affected by tagging (Figure 2, SOX2 example, and discussion ), the mechanistic investigation focuses more heavily on the C-terminal Ncaph2 example and AID leakage. This is perfectly acceptable given the scope, but perhaps a brief mention in the discussion regarding the challenges of predicting or identifying internal degrons affected by tags (if tags were to be placed internally as a suggested solution ) could be considered.

**We thank the reviewer for this comment and agree with their suggestion. We have added text to the discussion section of our revised submission (in red below) to highlight the possibility of internal degrons or other motifs at flexible internal loops.**

*“Alternatively, tags can be fused at internal flexible linker regions situated away from protein termini* (Adjobo-Hermans et al., 2011; Bendezú et al., 2015; Xu et al., 2024)*, although the possibility of internal degrons or other protein interaction motifs at these sites should also be considered”.*

Protocols and Data Availability:
• Protocols: The Materials and Methods section is exceptionally detailed and provides sufficient information for the experiments to be reproduced. This includes animal husbandry, generation of transgenic lines, tissue lysis, Western blotting, densitometry, cell culture (NSCs, ESCs, HeLa), molecular cloning, siRNA experiments, and bioinformatics for PSI predictions. The inclusion of specific reagents, concentrations, and equipment details is commendable. [e.g., 178, 181, 182, 184, 206, 212, 213, 217, 227, 228, 232, 238, 240, 252] The provision of Supplementary Tables for antibodies, nucleic acid sequences, and PSI predictions is excellent. Uncropped Western blots are also available.
• Data Availability: Original data appear to be within the manuscript and its Supporting Information files. Accession numbers for genes/proteins are implicitly handled via standard nomenclature and the PSI prediction pipeline uses UniProt. The software for PSI prediction is available on GitHub. Mouse lines are either previously described or noted for future detailed publication.

Adherence to Guidelines:
• The study appears to conform to relevant animal ethics guidelines, with approvals cited.
• The manuscript details should allow for robust assessment of the methodology.
Methodology Sufficiency for Reproduction: Yes, the methodological details provided are extensive and should allow other researchers to reproduce the experiments.
Software Availability: Yes, the machine learning model for PSI prediction is freely available on GitHub.
Organization and Clarity:
• The manuscript is very well organized, with a logical flow from introduction of the problem, presentation of results identifying tissue-specific effects, elucidation of underlying mechanisms, and finally, proposed solutions and broader implications.
• The writing is exceptionally clear, concise, and accessible even to those who may not be specialists in protein degradation or mouse genetics.
• Figures are generally clear and well-annotated, supporting the textual descriptions effectively.

Nomenclature and Abbreviations: Standard scientific nomenclature and abbreviations are used throughout the manuscript. Abbreviations are typically defined at their first use (e.g., AID, IAA, NSC).

Recommendation: This is a high-quality study presenting novel and significant findings that are of broad interest to the PLOS Genetics readership, particularly those working with transgenic models and protein tagging. The conclusions are strongly supported by rigorous experimental work. I recommend this manuscript for publication.

-----------

Reviewer #2: The manuscript by Taylor et al. presents a case study on the protein stability of tag-fused proteins in mice, with a particular focus on the condensin subunit NCAPH2. The authors previously reported a mouse line expressing NCAPH2-AID-Clover and found that the expression level of this protein was unaffected in hematopoietic cells (Macdonald et al., eLife, 2022). However, they observed that in some tissues, the expression levels were either higher or lower compared to the wild-type NCAPH2 protein (Fig. 1). Similarly, altered protein levels were detected when AID or HaloTag was fused to other proteins—such as DDX3, SOX2, and NCAPH—in a tissue-specific manner (Figs. 2 and S2). Based on these findings, the authors concluded that tag fusion can affect protein stability in a tissue-specific manner.

Regarding the underlying mechanism, the authors showed that NCAPH2-AID-Clover was degraded in the small intestine due to leaky degradation induced by endogenous indole-3-acetic acid (IAA) produced by gut microbes (Fig. 3). In contrast, in non-cycling neuronal cells in the brain, NCAPH2-AID-Clover levels were elevated, likely due to inefficient degradation of NCAPH2 in non-cycling cells (Fig. 4). To confirm whether the C-terminus of NCAPH2 contains a degron responsible for its reduced stability in non-cycling cells, the authors used a model ESC line expressing NCAPH2-GFP and found that the last 30 amino acids confer degron activity (Fig. 5).

Overall, this study was carefully conducted, and the presented data are convincing. Considering that tag-fusion strategies will be more frequently used in future studies with mice, this paper will be informative to anyone generating mice expressing a tag-fused protein.

**We are very grateful to the reviewer for their fair and thorough summary of our manuscript.**

Throughout the manuscript, the authors primarily focus on protein stability to explain the tissue-specific differences in expression. However, protein levels can also be influenced at the mRNA level, which the authors did not thoroughly investigate, except in the case of NCAPH2 (Fig. S3). Therefore, they should examine the mRNA levels of IFT81, DDX3, SOX2, and NCAPH from both the WT and tag-fused alleles (Fig. 2 and S2).

**We thank the reviewer for this comment. We agree that our original submission made no attempt to address whether other tagged mouse lines showed allele-specific differences in transcript levels that might differ in magnitude across tissues, and thus explain the differences observed at the protein level. To address this, we have now performed allele-specific qRT-PCR for two other transgenic lines (Sox2^Halo^ & Ncaph^AID:Clover^), comparing tissues where the ratio of tagged to untagged protein were relatively equal versus more skewed. In both cases, the ratio of transcripts encoding tagged to untagged proteins was comparable across tissues. The data are presented in an expanded Figure S3, and are consistent with our original finding for Ncaph2: the tissue-specific difference in the ratio of tagged to untagged gene product is not detected at the transcript level.**

**We note that the reviewer also explicitly requested allele-specific transcript quantification of *Ddx3x* and *Ift81* constructs. Unfortunately biological samples for RNA extraction were not available from these lines within the timeframe allowed for this revision. However, given that our manuscript did not claim that tags at these loci caused tissue-dependent differences at the protein level, we consider them to be lower priority.**

In Fig. 3C, the WB data were not ideal and difficult to interpret due to unequal protein loading. If the authors can show better blots, they should be presented.

**We agree that the protein loading is not perfectly even for the blot shown in Figure 3C. However, these assays are internally controlled; the experimental measurements are derived from the density ratio of tagged versus untagged bands from same blot. The loading control is not used for quantification so could theoretically be removed in this case, but we prefer to keep it for consistency with other figure panels. Unfortunately we do not have a better blot to show, but in our view the result is clear from the original.**

Reviewer #3: The manuscript by Taylor et al. describes a detailed exploration of the tissue-specific effectiveness and artifactual background in transgenic tagging of proteins across in vivo tissues. Although the theoretical possibility of such artifacts are well-understood, the careful empirical description of the propensity of these issues with C- and N-terminal tagging as described here will provide a highly useful illustration to the scientific community. In general I find the experiments well-described and carefully considered to address the key point of the manuscript.

**We are very grateful to the reviewer for their fair summary of our manuscript.**

My only suggestion would be to incorporate a bit more analysis in Fig S1, as as the authors note the tissue-specific presence of that non-specific band does make the interpretation of Fig 1 somewhat difficult. I think Fig. S1 is reasonable, but particularly as deep tissue RNA-seq data is available (and that band seems non-tag dependent), it should be easy to check that there isn’t a unique short isoform of NCAPH2 that might also explain this band (i.e., is expressed in heart/liver/kidney but not thymus / lung / spleen)? Particularly as a non-expected alternative splicing or alternative polyA site isoform would show similar patterns in Fig S1 (would lose expression off the 3’ tag, wouldn’t be altered by tag presence or absence, and might show decreased efficiency of siRNA knockdown depending on siRNA location – particularly as the siRNA pool only shows ~30% knockdown efficiency anyway).

**We thank the reviewer for this comment, and agree that our experiments in Figure S1 do not absolutely exclude the possibility that the lower band is a product of the Ncaph2 gene. However as we explain further below:**

**1) We and others have failed to find evidence that relevant gene products could exist**

**2) Even if they do, this would not significantly affect the claims in our manuscript**

1) **If the lower band is a Ncaph2 protein variant, it would have to use an alternative stop codon, because the band in question does not shift or change intensity in blots generated from tissue lysates from animals with 0 versus 2 alleles of the C-terminal fusion (Figure 1D, Figure S1A, S1B). Our laboratory has worked on the mouse Ncaph2 gene for over a decade** (Macdonald et al., 2022; Woodward et al., 2016)**, and published a study validating antibody probes against the protein** (Cutts et al., 2020)**. We are not aware of any evidence for C-terminal Ncaph2 protein variants. We draw the reviewer’s attention to a 2012 study which explicitly set out to characterise variant Ncaph2 gene products** (Theodoratos et al., 2012)**. Of 6 variant proteins, alternative start codons and internal splice sites were identified, but no evidence for alternative stop codons was reported. During the revision period we examined EST alignments in the UCSC mouse genome database looking for evidence of recurrent transcripts that contained alternative stop codons (e.g. via intron extension or retention towards the 3’ end of the gene). Consistent with the previous study** (Theodoratos et al., 2012)**, we did not find evidence for these transcripts.**
2) **If the lower band is a Ncaph2 protein variant, the implication is that the band densitometry in our original manuscript would have measured only proteins using the ‘canonical’ stop codon used for the fusion site, which is the only stop codon that is conserved across species. This would mean that any protein products from elusive Ncaph2 mRNAs containing an alternative stop codon were not measured in our assay. Even if this were true, we do not see how it would affect any of the claims in our manuscript regarding context-specific consequences of tag fusions.**

**To reflect our lack of absolute certainty that the lower band is not a product of the Ncaph2 gene, we have added the reference above and made the following change to the results text beginning on line 138:**

“Of note, the lowest molecular weight band on the western blot does not represent any known product of the *Ncaph2* gene (Theodoratos et al., 2012) because it is unaffected by the presence of the tag (Figure S1A), by auxin-inducible degradation (Figure S1B) or knockdown using pooled siRNAs (Figure S1C)”.

**Adjobo-Hermans, M. J., Goedhart, J., Weeren, L. van, Nijmeijer, S., Manders, E. M., Offermanns, S. and Gadella, T. W.** (2011). Real-time visualization of heterotrimeric G protein Gq activation in living cells. *BMC Biol.* **9**, 32–32.

**Bendezú, F. O., Vincenzetti, V., Vavylonis, D., Wyss, R., Vogel, H. and Martin, S. G.** (2015). Spontaneous Cdc42 Polarization Independent of GDI-Mediated Extraction and Actin-Based Trafficking. *PLoS Biol.* **13**, e1002097.

**Cutts, E. E., Taylor, G. C., Pardo, M., Yu, L., Wills, J. C., Choudhary, J. S., Vannini, A. and Wood, A. J.** (2020). A commercial antibody to the human condensin II subunit NCAPH2 cross-reacts with a SWI/SNF complex component. *Wellcome open Res.* **6**, 3.

**Macdonald, L., Taylor, G. C., Brisbane, J. M., Christodoulou, E., Scott, L., Kriegsheim, A. von, Rossant, J., Gu, B. and Wood, A. J.** (2022). Rapid and specific degradation of endogenous proteins in mouse models using auxin-inducible degrons. *eLife* **11**, e77987.

**Theodoratos, A., Wilson, L. O. W., Gosling, K. M. and Fahrer, A. M.** (2012). Splice variants of the condensin II gene Ncaph2 include alternative reading frame translations of exon 1. *FEBS J.* **279**, 1422–1432.

**Woodward, J., Taylor, G. C., Soares, D. C., Boyle, S., Sie, D., Read, D., Chathoth, K., Vukovic, M., Tarrats, N., Jamieson, D., et al.** (2016). Condensin II mutation causes T-cell lymphoma through tissue-specific genome instability. *Genes Dev.* **30**, 2173–2186.

**Xu, K., Li, Z., Mao, L., Guo, Z., Chen, Z., Chai, Y., Xie, C., Yang, X., Na, J., Li, W., et al.** (2024). AlphaFold2-guided engineering of split-GFP technology enables labeling of endogenous tubulins across species while preserving function. *PLOS Biol.* **22**, e3002615.
